# A cross sectional survey of urban Canadian family physicians' provision of minor office procedures

**DOI:** 10.1186/1471-2296-7-18

**Published:** 2006-03-19

**Authors:** Ian P Sempowski, Arne A Rungi, Rachelle Seguin

**Affiliations:** 1 Department of Family Medicine, Queens University, Kingston, Ontario, Canada; 2 Department of Orthopaedics, McMaster University, Hamilton, Ontario, Canada

## Abstract

**Background:**

A discordance exists between the proportion of Canadian family physicians that we expect should be able to perform minor office procedures and the actual provision of care. This pattern has not been extensively studied. The objective of this study was to determine the current patterns and obstacles relating to the provision of four minor office procedures by GP/FPs in a small city in Ontario, Canada. An additional goal was to determine the impact of the remuneration method on the provision of such services.

**Methods:**

A survey was mailed to all GP/FPs practising in Kingston, Ontario. The main outcomes measured in the study were work setting and remuneration method, current procedural practices with respect to four procedures, reasons for not performing procedures, current skill levels, and desire to upgrade.

**Results:**

Surveys were mailed to all 108 GP/FPs in the City of Kingston. Completed surveys were collected for 82 percent (89/108) and 10 were excluded leaving 79 eligible participants. The percentages of GP/FPs who reported performing the procedure were as follows: dermatological excision (63.3%), endometrial biopsy (35.4%), shoulder injection (31.6%), and knee injection (43.0%). The majority of GP/FPs who would not do the procedure themselves would refer to a specialist colleague rather than to another GP/FP. The top reason cited for not performing a specific procedure was "lack of up to date skills" followed by "lack of time". The latter was the only statistically significant difference reported between GP/FPs working in Family Health Networks and GP/FPs working in fee for service settings (26.7% vs 47.0%, χ^2 ^= 4.191 p = 0.041).

**Conclusion:**

A large number of Kingston, Ontario GP/FPs refer patients who require one of four minor office-based procedures for specialist consultation. Referral to other GP/FP colleagues appears underutilized. A perceived lack of up to date skills and a lack of time appear to be concerns. GP/FPs working in Family Health Networks were more likely to perform these procedures themselves. Further studies would clarify the role of changes in medical education, the role of continuing education, and the impact of different remuneration models.

## Background

In the Canadian health care system, general and family physicians (GP/FPs) play a large role in performing minor office procedures. GP/FPs have a broad base of general medical training that allows them to appreciate the indications, contraindications and non-procedural treatments for a wide array of conditions. With current stresses on the healthcare system, efficient use of available resources in meeting the needs of patients is crucial.

A British study found that dermatologic excision performed by general practitioners was more cost effective, patients were more satisfied with their treatment, and similar rates of complications occurred [1]. Another study looking at a broader range of minor procedures demonstrated high patient and referring physician satisfaction with no complications noted in the over 500 procedures performed [2]. While several studies have confirmed the cost effectiveness of general practitioners performing minor surgeries, Canadian data is limited [1-3].

The 2004 College of Family Physicians of Canada (CFPC) National Physician Survey reported that the proportion of certified (CCFP) FPs who self-reported performing skin excisions, joint injections and endometrial biopsies were 66.2%, 62.0% and 19.0% respectively [4]. Other studies have confirmed that FPs in rural areas are more likely to perform these procedures than their urban counterparts [5-7]. Male physicians have also been shown to have higher rates than female physicians [8].

In a recent small Canadian research study, 100% of the 36 respondents listed dermal excision as being a core procedure expected of all graduating family physicians. In this study, 89% currently perform this procedure themselves and the 2004 National Physician Survey data for this procedure was 66.2% [4,5].

A number of factors contribute to the apparent discordance between expected and actual procedure provision rates. In a study of Alberta family physicians 91% of respondents reported having learned the procedures in medical school or residency with a minority learning through clinical practice or continuing medical education[9]. New graduates may simply not feel confident in their technical skills due to a lack of exposure in medical school or residency. The widespread shortages of GP/FPs may create practice environments where the doctors are just "too busy". The effort and cost of buying and maintaining equipment may also be an issue. Finally, there may be financial remuneration issues.

The impact of Ontario primary care reform initiatives such as Family Health Networks (FHNs) and Family Health Groups (FHGs), in contrast to the traditional fee for service (FFS) models is not clear. FHNs are groups of physicians who roster patients, receive a capitated payment for their care and are eligible for a number of financial incentives for achieving targets for preventative health screening and provision of a broad scope of services. FHGs are groups that require rostering and receive limited bonuses but are still essentially FFS.

The primary objective of this study was to determine the current practices of Kingston GP/FPs with respect to minor office procedures. A secondary objective was to determine what self reported obstacles prevented a higher provision and what work setting factors might be associated with a higher rate. Finally, we wanted to determine whether physicians in FHNs working outside of a FFS model were more or less likely to perform the selected procedures.

## Methods

A survey was developed and a draft was pilot tested with a small group of academic family physicians who commented on the measure's ease of use, clarity, completeness and relevance. These comments were incorporated into the final version. The Queen's University Research Ethics Board approved the survey and study. All GP/FPs identified in the city of Kingston as practicing family medicine or general practice through the phone directory and the local professional association were mailed a survey package. Kingston, Ontario, Canada is a small city with a population of 120,000 people. It has a tertiary care hospital and medical school. Survey packages including a questionnaire, cover letter, and postage-paid return envelope were mailed to participants between November 2003 and January 2004. The Dillman method of two follow-up mailings to non-responders was modified to only include one follow-up mailing was carried out because the response rate was initially high and a large number of non- responders specifically expressed a lack of interest in completing the survey [10]. Physicians were excluded from the final analysis if family medicine practice accounted for less than 30 percent of their clinical time. The rationale was to exclude physicians who had chosen to practice in focused areas distinct from primary care. The absolute frequency and percentages were calculated and chi square analysis was calculated and tested for statistical significance using SPSS software version 11 [11].

Potential study procedures were initially chosen based upon the list of core procedures taught in the Queens University Family Medicine program. This list was cross-referenced against other published lists [6,7]. We then polled 20 academic and non-academic practitioners regarding their current practices. We wanted to examine procedures that were included on the core teaching list but appeared to be least uniformly performed in the pilot study. We narrowed the selection down to four procedures: dermatological excision or punch biopsy, endometrial biopsy, shoulder joint injection – intra-articular or subacromial bursa, and knee injection or aspiration. Published surveys of both family physicians and family medicine program directors, showed that 75–99 percent of respondents considered the four procedures to be core procedures for family physicians [12,13].

The survey asked a series of questions in multiple-choice format. The first outcome measure focused on *work setting *ie. FHN, FHG, hospital clinic, private group practice or solo practice. *Current practice *with respect to the four selected procedures i.e. perform yourself, refer to a family medicine colleague, refer to a specialist, or do not see applicable patients. The next outcome measure was *reasons for not performing the procedure*, as outlined in Table 1. Finally, we asked whether the physician had an interest in *upgrading their skills *with respect to each of the four procedures.

**Table 1:** Obstacles identified by Kingston family physicians that did not perform the study procedures in their practice

**Reasons for not doing procedure**	**Dermatological Excision N = 29**	**Endometrial Biopsy N = 53**	**Shoulder injection N = 54**	**Knee injection or aspiration N = 45**
**Lack of up to date skills**	51.7%	59.6%	70.4%	75.6%
**Do not like procedure**	13.8%	9.8%	18.5%	15.6%
**Do not see patients with indications**	0.0%	7.8%	9.3%	8.9%
**Complexity**	3.4%	3.9%	13.0%	13.3%
**Cost of equipment**	31.0%	17.6%	7.4%	8.9%
**Time**	55.2%	45.1%	37.0%	42.2%
**Fees too low**	24.1%	21.6%	14.8%	17.8%
**Easier to refer**	24.1%	27.5%	16.7%	17.8%

## Results

Surveys were mailed to 108 GP/FPs and 82 percent (n = 89) were returned completed. Ten of the respondents were excluded from the analysis because less than 30 percent of their time was spent practicing family medicine, leaving 79 GP/FPs in the study group. The majority of those excluded were trained family physicians working as full time emergency room doctors. Physicians working in FHNs represented 25.3 percent of the total with the remaining proportion compensated by FFS. The latter group included FHG physicians (45.6% of total), other group practice physicians (13.9%), solo practitioners (11.4 %), and hospital clinic practices (3.8%).

The overall self reported percentages of GP/FPs who performed the procedures were as follows; dermatological excision (63.3%), endometrial biopsy (35.4%), shoulder injection (31.6%), and knee injection (43.0%). Of those who did not perform the procedure, a minority (range of 3.8–17.8% for the 4 procedures) of GP/FPs in this study self reported referring to a family medicine colleague rather than to a specialist (range of 31.2–52.1%).

When comparing GP/FPs working in FHNs to GP/FPs working in other settings, there was a significant increase in FHN doctors' provision for endometrial biopsy (χ^2 ^= 10.4 p = 0.01) and a trend towards a difference for shoulder injection (χ^2 ^= 7.300 p = 0.06). However, there were no significant differences in current practices between the two groups for dermatological excision and knee injection or aspiration.

Lack of up to date skills and time were consistently the top two cited obstacles for GP/FPs when performing procedures. The percentages of GP/FPs who cited each of the various obstacles are outlined in Table 1. Of the subgroups of GP/FPs who did not perform each of the procedures, identifying "lack of up to date skills" ranged from a low of 51.7 percent of GP/FPs for dermatological excision to a high of 75.6 percent of GP/FPs for knee injection or aspiration. Identifying "time" ranged from a low of 37.0 percent of GP/FPs for shoulder injections to a high of 55.2 percent of physicians for dermatologic excisions.

A comparison of physicians working in FHNs to physicians working in all other practice types combined is outlined in Table 2. The proportion of FHN respondents who cited "time" as a barrier was consistently less than in other practice types. When all four procedures were combined, "time" was the only variable which demonstrated a significant difference between physicians working in FHNs and physicians working in other settings (26.7% vs. 47.0%, χ^2 ^= 4.191 p = 0.041). Other variables showed trends towards differences; FHN respondents were less likely to cite "do not like procedures" (3.3% vs. 16.5%, χ^2 ^= 3.636 p = 0.057) as a reason for not performing the procedure. The concern that "fees are too low" was also lower in the GP/FPs working in a FHN (6.7% vs. 21.5%, χ^2 ^= 3.560 p = 0.059). For all four procedures, a small proportion (range of 0%-9.3%) of physicians reported, "Do not see patients with indications."

**Table 2:** A comparison of the cited reasons for not performing one or more of the procedures for FHN doctors and non – FHN doctors (all other practice types combined)

**Reasons for not doing procedure**	**Percentage of FHN doctors who cited this as a reason for** ** not performing one or more of the four procedures**	**Percentage of non-FHN doctors**	Statistical significance * p <.05
**Lack of up to date skills**	61.4%	71.7%	NS
**Do not like procedure**	3.3%	16.5%	χ^2 ^= 3.636 p = 0.057
**Do not see patients with indications**	4.0%	4.6%	NS
**Complexity**	7.5%	10.2%	NS
**Cost of equipment**	18.5%	24.8%	NS
**Time**	26.7%	47.0%	χ^2 ^= 4.191 p = 0.041 *
**Fees too low**	6.7%	21.5%	χ^2 ^= 3.560 p = 0.059
**Easier to refer**	18.5%	23.8%	NS

The survey also asked about current skill levels and the desire of the physicians to upgrade their skills. Dermatological excision showed a high skill rate with 68.4 percent of GP/FPs confident in their skills, 24.1 percent of GP/FPs would attend a workshop, and only 11.4 percent of GP/FPs lacked interest in performing the procedure. The other three procedures were all similar statistically with a range 30.4 – 39.2 percent of GP/FPs reporting confidence with their skill; 26.6 – 32.2 percent reporting they would attend a workshop; and 32.9 – 34.2 percent reporting no interest in performing the procedure.

## Discussion

A significant number of GP/FPs in practising in Kingston, Ontario report that they do not perform the four selected minor office procedures. It is unlikely that GP/FPs do not see patients with indications for these procedures. The two main stated reasons for this pattern were "lack of up do date skills", and "time" with the concern regarding time being less prevalent in the FHN physicians. Dermatological excision was the only procedure identified in the survey that the majority of GP/FPs (63.3%) would perform themselves. With respect to endometrial biopsy, shoulder injection and knee injection, the majority of respondents would refer to a specialist colleague.

Naismith et. al. described the importance of teaching minor surgical procedures as an essential component of the family medicine residency curriculum [14]. A systematic, organized and documented procedural skills curriculum at the undergraduate and residency level is required. The creation of national recommendations such as those recently published by the CFPC procedural skills working group should help to create a national standard [4]. For physicians whose skills require updating there does appear to be support for targeted workshops (for all but dermatologic excision) based upon this study. Britain experienced a dramatic drop in referral of minor surgical procedures to specialists after completion of educational programs [15]. Furthermore, increased provision of procedures at the primary care level has not been associated with a decline in the quality of care [16]. For those who do not wish to perform the procedures referring to another GP/FP who does perform procedures is an option which remains relatively unexploited. Only a minority (range of 3.8–17.8%) of GP/FPs in this study self reported referring to a family medicine colleague rather than a specialist (range of 31.25–52.1%).

"Time" was the second highest cited reason for not performing the study procedures. In a fee for service (FFS) model, time and remuneration are clearly linked. In our study FHN doctors appeared to report lack of time less frequently than their fee for service counterparts. FHN remuneration may allow practitioners to be less concerned with time and volume of patients seen. Alternatively, those who have chosen to be in a FHN may be inherently different with respect to their skills or attitudes. This may be illustrated by our trend toward FHN physicians having a lower rate of "do not like procedures".

The results of this study can only be extrapolated to other similar small Canadian urban settings. In smaller towns and in rural areas it is likely that the provision of these four procedures would be higher [6,7]. This study is limited in its power by the small number of participants. Statistical significance was difficult to achieve in the sub groups of those who do not perform each of the four procedures. Finally, physician self reported practice patterns may be less accurate than an objective measure such as health plan billings. Further studies should focus on medical education and continuing education as well as the impact of different remuneration models.

## Conclusion

Many family physicians in Kingston refer patients requiring minor office procedures for specialist consultation. Treating patients at the primary care level can be more timely and cost effective. "Lack of up to date skill" and "time" were the top reasons cited for not performing the procedures. Excision of dermatologic lesions was performed at a higher rate than the other three study procedures. A significantly lower percentage of physicians working in FHNs as opposed to fee for service models identified "time" as being a reason for not performing the procedures. Only a minority of GP/FPs did not see patients with the indications or were not interested in performing the procedures, suggesting that enhanced educational opportunities may be beneficial. Further studies are needed to examine the role of changes in medical education, the role of continuing education, and the impact of different remuneration models.

## Abbreviations

FFS fee for service

FHG family health group

FHN family health network

GP general practitioner

FP family physician

CFPC College of Family Physicians of Canada

## Competing interests

The author(s) declare that they have no competing interests.

## Authors' contributions

IPS and AAR contributed equally to this work through study design, data collection, analysis, and writing.

RMS assisted in study and survey design as well as data collection, analysis and editing.

## Pre-publication history

The pre-publication history for this paper can be accessed here:


